# State marijuana laws and opioid overdose mortality

**DOI:** 10.1186/s40621-019-0213-z

**Published:** 2019-09-02

**Authors:** Stanford Chihuri, Guohua Li

**Affiliations:** 1 Center for Injury Epidemiology and Prevention, Columbia University Irving Medical Center, 622 West 168th St, PH5-505, New York, NY 10032 USA; 2 Department of Anesthesiology, Columbia University College of Physicians and Surgeons, 622 West 168th St, PH5-505, New York, NY 10032 USA; 3 Department of Epidemiology, Columbia University Mailman School of Public Health, 622 West 168th St, PH5-505, New York, NY 10032 USA

**Keywords:** Cannabis, Drug overdose, Drug policy, Marijuana legalization, Opioid epidemic

## Abstract

**Background:**

The opioid epidemic in the United States is a national public health crisis. In recent years, marijuana legalization has been increasingly adopted by state governments as a policy intervention to control the opioid epidemic under the premise that marijuana and opioids are substitutive substances. The purpose of this systematic review is to synthesize the empirical evidence regarding the impact of state marijuana laws on opioid overdose mortality and other opioid-related health outcomes.

**Method:**

A comprehensive search of the research literature in 18 bibliographic databases returned 6640 records, with 5601 abstracts reviewed, 29 full text articles screened for eligibility, and 16 eligible studies included in the systematic review. Comprehensive Meta-Analysis software was used to generate summary estimates, forest plots, funnel plots, and heterogeneity statistics.

**Results:**

Of the 16 eligible studies, 4 assessed the association of state marijuana law status with opioid overdose mortality, 7 with prescription opioids dispensed, and the remaining with nonmedical use and opioid-related hospitalizations. Random effects modeling based on pooled data revealed that legalizing marijuana for medical use was associated with a statistically non-significant 8% reduction in opioid overdose mortality (95% confidence interval: − 0.21 to 0.04; *p* = 0.201) and a 7% reduction in prescription opioids dispensed (95% confidence interval: − 0.13 to − 0.01; *p* = 0.017). Legalizing marijuana for recreational use was associated with an additional 7% reduction in opioid overdose mortality in Colorado and 6% reduction in opioid prescriptions among fee-for-service Medicaid and managed care enrollees.

**Conclusions:**

Legalizing marijuana might contribute to a modest reduction in opioid prescriptions. Evidence about the effect of marijuana legalization on opioid overdose mortality is inconsistent and inconclusive. If any, the effectiveness of state marijuana laws in reducing opioid overdose mortality appears to be rather small and limited to states with operational marijuana dispensaries. It remains unclear whether the presumed benefit of legalizing marijuana in reducing opioid-related harms outweighs the policy’s externalities, such as its impact on mental health and traffic safety.

## Introduction

Drug overdose is the leading cause of injury mortality in the United States (Ahmad et al. 2018; Centers for Disease Control and Prevention 2019a; Rudd et al. 2016a; Scholl et al. 2018). In 2017, more than two-thirds of the 70,237 drug overdose deaths involved an opioid (Hedegaard et al. 2018). The opioid epidemic has gone through three phases. The first phase started with the introduction of OxyContin in 1996 and was fueled by overconsumption of prescription opioids (Centers for Disease Control and Prevention 2019b; 2018a; Compton and Volkow 2006; Kolodny et al. 2015). The second phase was marked by a sharp increase in heroin-related overdose deaths between 2010 and 2015, presumably because heroin became more affordable, potent and accessible than prescription opioids (Bipartisan Policy Center 2019; Centers for Disease Control and Prevention 2018a; Cicero et al. 2014; Compton et al. 2016; Dasgupta et al. 2018). Finally, the third phase started in late 2013 and continues to present day, characterized by the steady increase in overdose deaths involving illicitly manufactured fentanyl and analogs (Centers for Disease Control and Prevention 2019b; Cicarrone 2017; Rudd et al. 2016b; Seth et al. 2017). In response to the continuing increase in overdose mortality, the US federal government declared the opioid epidemic a national public health emergency in October 2017.

Although the current phase of the opioid epidemic is primarily driven by illicit fentanyl and analogs, prescription opioids continue to play a significant role, contributing to more than 35% of the overall overdose mortality (Centers for Disease Control and Prevention 2019b). Between 1999 and 2017, prescription opioid overdose claimed about 218,000 lives in the United States (Centers for Disease Control and Prevention 2018b, 2019b; Scholl et al. 2018). Among patients with chronic pain who take prescription opioids, 21 to 29% misuse them and 8 to 12% develop an opioid use disorder (Hedlund and Macek 2018). In addition, misuse of prescription opioids may progress to heroin use (Cicero et al. 2014; Jones et al. 2013; Rudd et al. 2016b) and increase the risk of being involved in fatal motor vehicle crashes (Chihuri and Li 2017a, 2019; Chihuri and Li 2017b; Li and Chihuri 2019). Although the annual opioid prescribing rate has declined in recent years, it remains high, at about 0.6 prescription per capita (Centers for Disease Control and Prevention 2019b). The most common prescription opioids involved in overdose deaths are oxycodone, hydrocodone, and methadone (Hedegaard et al. 2018).

To address the opioid epidemic, state governments are increasingly moving toward legalizing marijuana for medical or recreational use under the premise that marijuana represents a less harmful alternative to prescription opioids for chronic pain management. Currently, 34 states and the District of Columbia have legalized marijuana for medical use among those with qualifying health conditions, and 10 states and the District of Columbia have legalized marijuana for recreational use among those 21 years of age and older (National Conference of State Legislatures 2019). However, marijuana remains a Schedule I substance under the federal law in the United States. Marijuana has been found to be an alternative therapy among patients with neuropathic pain (Andreae et al. 2015; Ellis et al. 2009; Ware et al. 2010; Wilsey et al. 2013), treatment-resistant epilepsy (Devinsky et al. 2016; Friedman and Devinsky 2015), chronic pain (Haroutounian et al. 2016; Nugent et al. 2017; Savage et al. 2016; Ware et al. 2015; Whiting et al. 2015; Wilkinson et al. 2016), multiple sclerosis (Rog et al. 2005), and diabetic neuropathy (Wallace et al. 2015). In 2017, the National Academies of Sciences, Engineering, and Medicine concluded that marijuana is an effective treatment for chronic pain among adults (National Academies of Sciences Engineering and Medicine 2017). Since 2014, there have been several studies assessing the impact of state marijuana laws, particularly medical marijuana laws (MMLs), on opioid-related harms. Although a recent narrative review suggests that MMLs could reduce opioid overdose mortality and healthcare costs (Vyas et al. 2018), marijuana legalization as a policy intervention to control the opioid epidemic remains controversial because no consensus has emerged on the health consequences of marijuana use (Bradford et al. 2018; Olfson et al. 2018; Phillips and Gazmararian 2017; Powell et al. 2018; Stith et al. 2018; Wen and Hockenberry 2018). This systematic review aims to provide an updated assessment of empirical research evidence pertaining to the impact of state marijuana laws on opioid overdose mortality and other opioid-related health outcomes, such as opioid prescriptions and opioid-related hospitalizations.

## Methods

We conducted a systematic review of published and grey literature and performed meta-analyses for the associations of MMLs with opioid overdose mortality and opioid prescription dispensed by following the Preferred Reporting Items for Systematic Reviews and Meta-Analyses (PRISMA) and Meta-Analyses of Observational Studies in Epidemiology (MOOSE) guidelines (Moher et al. 2009; Stroup et al. 2000).

### Eligibility

Studies were eligible for inclusion if they: 1) were based on population data and research designs ensuring that the exposure (i.e., state marijuana laws) preceded prescription opioid-related outcome; 2) had an appropriate comparison group (i.e., non-MML states or pre-MML time periods); 3) presented quantitative data; and 4) were published in the English language. Qualitative studies, commentaries, opinion pieces, letters, editorials, and reviews were excluded. Also excluded were studies that focused on illicit opioids, surveys on opioid use, and studies conducted outside of the United States. No date restrictions were applied.

### Search strategy, data sources and extraction

During March 10–15, 2019, we searched the following 18 electronic databases: PubMed (1966-present), Google Scholar, EMBASE (Ovid) (1980-present), Health and Psychosocial Instruments (1985-present), The Cochrane Central Register of Controlled Trials (1993-present), Database of Cochrane Systematic Reviews (1993-present), American Psychological Association PsycInfo (1967-present), The Joanna Briggs Institute EBP Database (1996-present), Scopus (1960-present), Transport Research International Documentation (TRID)(1970-present), American College of Physicians Journal Club (1967-present), the Cumulative Index to Nursing and Allied Health (1982-present), EBM Reviews (1980-present), Database of Abstracts of Review of Effectiveness (1982-present), Web of Science (1900 to present), MEDLINE (1946-present), MELVYL (the online catalog of the University of California library system) (1970-present), and SafetyLit (1995-present). A further search was conducted by manually reviewing reference lists of identified eligible articles. These databases were searched using outcome keyword ‘opioid’, exposure keyword ‘marijuana law’ and corresponding MeSH terms. MeSH terms included [(analgesic or opiate or pain medication or pain treatment) and (overdose or mortality or death or morbidity or hospitalization or substance use disorder or addiction or admission or prescription or dose or dosage or morphine equivalent or misuse, abuse, nonmedical use, illegal use) and (marijuana or cannabis or THC) and (law or policy or legislation or implementation or statute or dispensaries)]. Studies that were possibly eligible were reviewed in full text. Information on primary author, publication year, states, study population including comparison groups, study design, outcomes assessed, data sources, covariates, and key findings were abstracted from included studies. Both authors independently verified the data abstracted from identified studies and resolved discrepancies through discussion and consensus.

### Quality assessment, data synthesis, and analysis

We used the Newcastle-Ottawa Scale (NOS) for assessing nonrandomized studies to evaluate the quality of the studies included as suggested by the Cochran Collaboration (Higgins and Green 2011; Wells et al. 2015). The NOS scales range from one to nine with higher scores indicating better quality. In addition, studies are assessed as good quality if they score three or four on selection, one or two on comparability, and two or three stars on outcome. Studies are assessed as fair quality if they score two on selection, one or two on comparability, and two or three stars in outcome. Finally, studies are assessed as poor quality if they score zero or one on selection, or zero on comparability, or zero or one on outcome. Standard Q and I^2^ statistics were used to assess heterogeneity (Borenstein et al. 2009). Summary estimates from the random effects models were used where significant heterogeneity was present (Borenstein et al. 2019). Data abstracted from each study were used to generate summary estimates, forest plots, funnel plots, heterogeneity statistics, and weights for each study using the Comprehensive Meta-Analysis software (Borenstein et al. 2005).

## Results

### Sample and study characteristics

**Fig. 1:**
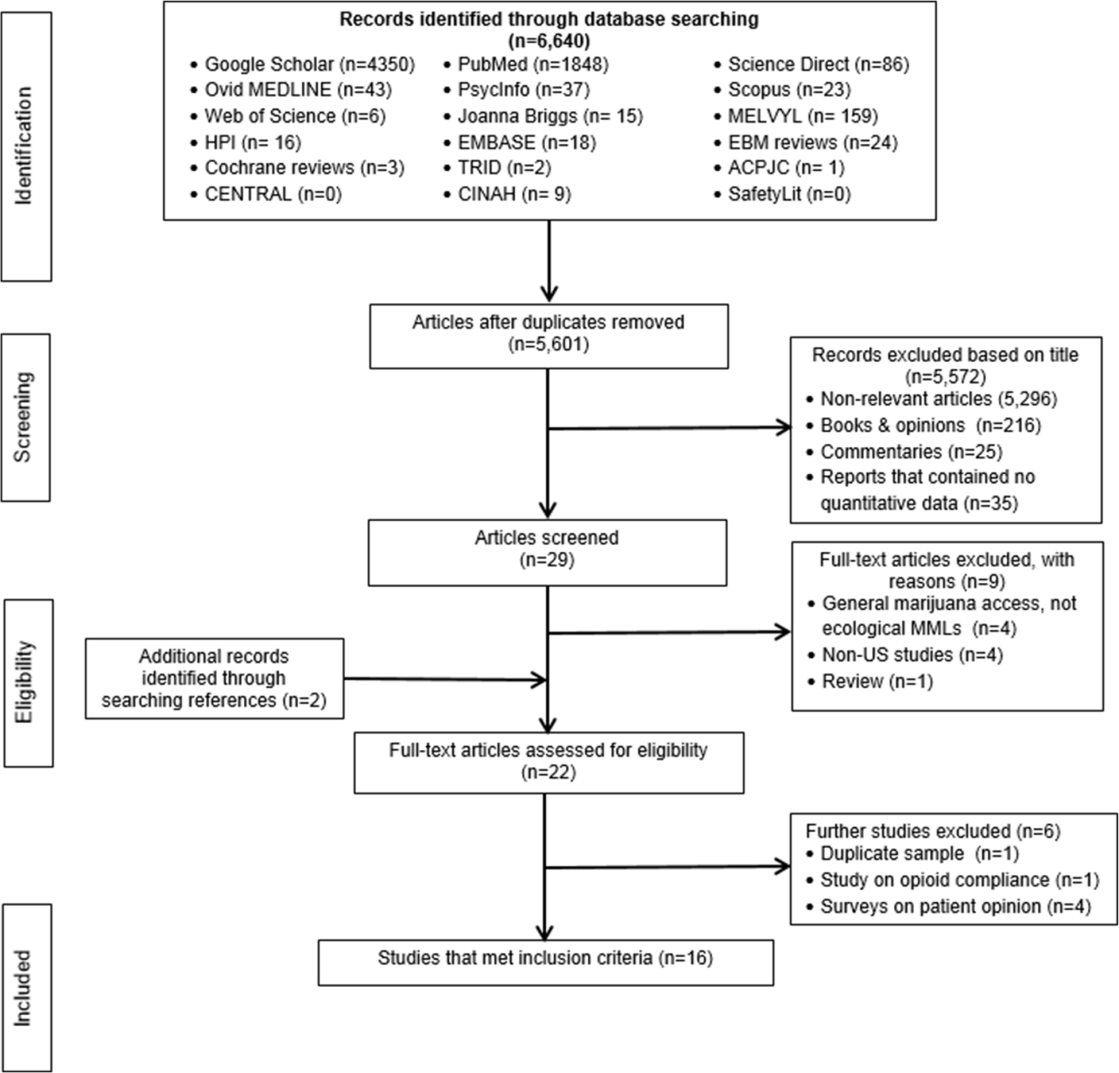
Flowchart of identification, screening, eligibility review and selection of studies included in the systematic review on the association of MMLs and prescription opioid-related outcomes in the U.S. Adapted from (Moher et al. 2009)

The initial comprehensive database search identified 6640 records. After duplicates were removed, 5601 titles were screened for eligibility. Of these, a total of 5572 were excluded because they were: 1) irrelevant to the research question (*n* = 5, 296); 2) book excerpts or opinion pieces (*n* = 216); 3) commentaries (*n* = 25); or 4) reports that contained no quantitative data (*n* = 35). Of the remaining 29 records, 9 articles were excluded upon full text screening for reasons such as absence of MML evaluation, being conducted outside of the United States, and lack of quantitative data (Fig. 1). The full text articles of the remaining 20 records were reviewed for eligibility and 2 additional articles were identified through a manual search of the references. Both authors then agreed to exclude four survey-based studies on patient opinions regarding substitution of medical marijuana for opioid medications (Boehnke et al. 2016; Corroon et al. 2017; Reiman et al. 2017; Sexton et al. 2016), and one study on patient opioid compliance (Lo et al. 2019), leaving 17 eligible studies (Fig. 1). Another study (Vigil et al. 2017) was excluded because the data were included in a separate eligible study by the same research team (Stith et al. 2018). Overall, 4 studies presented results regarding the impact of state MMLs on opioid overdose mortality (Bachhuber et al. 2014; Phillips and Gazmararian 2017; Powell et al. 2018; Smart 2016), 7 on opioid prescriptions dispensed (Bradford and Bradford 2017; Bradford and Bradford 2016; Bradford et al. 2018; Liang et al. 2018; Powell et al. 2018; Stith et al. 2018; Wen and Hockenberry 2018), 3 on nonmedical use or abuse of prescription opioids (Cerda et al. 2018; Shi 2017; Wen et al. 2015), and two on prescription-opioid related hospitalizations (Powell et al. 2018; Shi 2017). In addition, 1 study assessed the effect of legalizing marijuana for recreational use on opioid overdose mortality (Livingston et al. 2017), 1 study assessed the effect of legalizing marijuana for recreational use on opioid prescriptions (Wen and Hockenberry 2018) and 1 study assessed the association between state MMLs with prescription opioid positivity among fatally injured drivers (Kim et al. 2016). We performed two meta-analyses, one based on pooled data from the 4 studies examining the association of state MMLs with opioid overdose mortality and the other based on pooled data from the 7 studies assessing the impact of state MMLs on opioid prescriptions dispensed. One study (Powell et al. 2018) contributed data to both meta-analyses. Table 1 summarizes the 16 studies included in the review. These studies were published between 2014 and 2018, including 1 dissertation (Smart 2016) and 15 peer-reviewed articles.

**Table 1:** Characteristics of studies evaluating the association between MMLs and opioid- related outcomes in the US

Outcome	Author(s), year	Study time period	State	Study subjects	Study design/analysis	Opioid-related outcome measure	Outcome data source	Covariates	Key findings	^a^Quality score
Mortality	Powell et al. 2018	1992–2013	All states	Subjects from 24 states with MMLs compared with those from non-MML states	Difference-in-differences	Prescription opioid-related mortality	National Vital Statistics System	Age, % male population, unemployment rate, alcohol taxes, log of population	MMLs were associated with a 4.8% reduction in opioid overdose mortality	8
	Phillips and Gazmararian 2017	2011–2014	All states and D.C	US population during the study period	Ecological analysis	Age-adjusted opioid-related mortality	Multiple Cause of Death database, CDC WONDER	State urban population, state disability rates, education, annual unemployment rates	MMLs were associated with a 1.7% increase in opioid-related mortality	8
	Smart 2016	1999–2013	48 states	Subjects who died from prescription opioid overdose	Poisson regression	Prescription opioid-related mortality	Multiple Cause of Death database, CDC WONDER	Age, % male population, unemployment rate, alcohol taxes, population	MMLs were associated with a 7.2% reduction in opioid overdose mortality.	7
	Bachhuber et al. 2014	1999–2010	All states	Subjects from 13 MML states; 3 states with MML enacted prior to the study and 10 enacted during the study period	Time-series analysis	Age-adjusted prescription opioid overdose death rate	Multiple Cause of Death database, CDC WONDER	PDMP status, laws requiring identification before dispensing, state oversight, unemployment rates	MMLs were associated with a 24.8% reduced state-level prescription opioid overdose mortality rates	8
	Livingston, 2017	2000–2015	Colorado	Subjects who from opioid overdose in Colorado (recreational marijuana law), Nevada (MML), and Utah (no MML)	Time-series analysis	Opioid-related mortality	Multiple Cause of Death database, CDC WONDER	PDMP status, trends in opioid-related deaths in Nevada and Utah	Recreational marijuana was associated with a 6.5% reduction in opioid-related deaths	6
Prescriptions dispensed	Bradford et al. 2018	2010–2015	All states	All fee-for-service Medicare Part D prescriptions for all opioids	Multi-level regression	Daily opioid doses prescribed (in millions) per state-year	Medicare Part D Prescription Drug Event Standard Analytic File	PDMP status, Physician market competition, % below FPL, total population, % enrolled in Medicare, % in Medicare Advantage plans, state fixed effects	MMLs of any type were associated with a decrease of 8.5% daily opioid doses prescribed per state-year	7
	Liang et al. 2018	1993–2014	All states	Patients enrolled in fee-for-service Medicaid programs	Time-series analysis	Opioid prescriptions per quarter year per 100Medicaid enrollees	Medicaid State Drug Utilization Data	PDMP, Medicaid expansion, household income, active physicians per 1000 population, % residents with household income below FPL, unemployment rate	MMLs were not associated with Schedule II opioid prescriptions dispensed. However, MMLs were associated with 15% decrease in Schedule III opioid prescriptions	8
	Powell et al. 2018	1992–2013	All states	Subjects from 24 states with MMLs compared with those from non-MML states	Difference-in-differences	Opioid prescriptions filled	National Vital Statistics System	Age, % male population, unemployment rate, alcohol taxes, log of population	MMLs were associated with a 3.3% increase in opioid prescriptions	8
	Stith et al. 2018	2010–2015	New Mexico	83 chronic pain patients enrolled in New Mexico medical marijuana program; 42 non-enrolled patients	Retrospective cohort	Schedule II drug prescriptions	Prescription drug monitoring program records	Age, sex	Enrolling in the medical marijuana program was associated with a 4% reduction in Schedule II drug prescriptions filled.	6
	Wen and Hockenberry 2018	2011–2016	All states	All fee-for-service Medicaid and managed care enrollees	Difference-in-differences	Opioid prescriptions filled	Medicaid State Drug Utilization Data	Age, sex, PDMP status, Pain medication laws, poverty rates, household income, unemployment status, number of Medicaid prescriptions	MMLs were associated with a 5.9% reduction in the rate of opioid prescriptions and legalizing creational marijuana was associated with a 6.38% reduction in the rate of opioid prescriptions.	7
	Bradford and Bradford 2017	2007–2014	All states	All fee-for-service Medicaid prescriptions covering 9 clinical areas of prescription drugs for which MM could be an alternative	Difference-in-differences	Daily doses of prescriptions for pain medications per quarter year per Medicaid enrollee	Medicaid State Drug Utilization Data	Physicians per capita, poverty rate, unemployment rate, state total population, median income, PDMP status	MMLs were associated with an 11% reduction in daily doses of prescriptions for pain medications	7
	Bradford and Bradford 2016	2010–2013	All states	All fee-for service Medicare Part D prescriptions covering 9 clinical areas of prescription drugs for which MM could be an alternative	Difference-in-differences	Daily doses of prescriptions for pain medications filled per physician per year	Medicare Part D Prescription Drug Event Standard Analytic File	Physicians per capita, county unemployment rate, county total population, racial composition, SES, county mortality rate, physician sex	MMLs were associated with a 14.3% reduction in daily doses of prescriptions for pain medications filled per physician per year	6
Hospitalizations	Powell et al. 2018	1992–2013	All states	Subjects from 24 states with MMLs compared with those from non-MML states	Difference-in-differences	Prescription opioid-related hospitalizations	National Vital Statistics System	Age, % male population, unemployment rate, alcohol taxes, log of population	MMLs were not associated with prescription opioid-related hospitalizations	8
	Shi 2017	1997–2014	27 states	Subjects who were hospitalized in states that participated in the State Inpatient Databases	Time-series analysis	Opioid pain reliever overdose hospitalizations per state per year	State Inpatient Databases, Healthcare Cost Utilization Project	State population size, unemployment rate, median household income, beer tax per gallon, health uninsured rate	MMLs was associated with a 13% reduction related to opioid pain reliever overdose hospitalizations	8
Non-medical use	Cerda et al. 2018	1991–2015	48 states	8th, 10th, and 12th graders	Difference-in-differences	Self-reported nonmedical use of prescription opioids	National Monitoring the Future annual survey	Grade, age, sex, race/ethnicity, SES, students per grade, type pf school, urbanicity, percent of state population that was male, White and aged 10–24 years or 25 years and older, alcohol and cigarette taxes	MML was associated with a 0.3% reduction, and a 0.3% increase in nonmedical use of prescription opioids among 10th and 12th graders respectively. The was no change among 11th graders	8
	Shi 2017	1997–2014	27 states	Subjects who were hospitalized in states that participated in the State Inpatient Databases	Time-series analysis	Opioid pain reliever abuse or dependence –related hospital discharges per state per year	State Inpatient Databases, Healthcare Cost Utilization Project	State population size, unemployment rate, median household income, beer tax per gallon, health uninsured rate	MMLs was associated with a 23% reduction in opioid pain reliever abuse or dependence-related hospitalization	7
	Wen et al. 2015	2004–2012	10 states	Civilian, non-instutionalized subjects aged 12 years and older	Probit regression	Non medically used prescription pain medications	National Survey on Drug Use and Health	Age, gender, race/ethnicity, health status, smoking status, health insurance status, family income, urbanicity, marital status, education attainment, college enrollment, employment status, state unemployment rate, average personal income, median household income, beer tax per gallon	MML was not associated with any significant change in the rate of nonmedical prescription pain medications use	8
Opioid positivity among fatally injured drivers	Kim et al. 2016	1999–2013	18 states that tested for alcohol and drugs in at least 80% of all fatally injured drivers	Fatally injured drivers who died within 1 h of crash	Multi-level logistic regression	Opioid positivity	Fatality Analysis Reporting System	Age, sex, PDMP status, blood alcohol concentration	MMLs were associated with a reduction on opioid positivity among 21–40 year old fatally injured drivers (OR = 0.50 95%ci = 0.37–0.67)	7

^a^Threshold assessment: Good quality: 3 or 4 stars in selection domain AND 1 or 2 stars in comparability domain AND 2 or 3 stars in outcome/exposure domain; Fair quality: 2 stars in selection domain AND 1 or 2 stars in comparability domain AND 2 or 3 stars in outcome/exposure domain; Poor quality: 0 or 1 star in selection domain OR 0 stars in comparability domain OR 0 or 1 stars in outcome/exposure domain

### Study quality

All studies used appropriate statistical methods and adjusted for some covariates such as demographic characteristics and potential confounders such as prescription drug monitoring programs (PDMPs) and other statewide policies (Table 1). Overall, 13 studies were of good quality and 3 of fair quality (Bradford and Bradford 2016; Livingston et al. 2017; Stith et al. 2018), with an average score of 7.4 out of 9 (range from 6 to 8) on the Newcastle-Ottawa Scale. Studies with lower scores analyzed data from a single state and therefore were of limited generalizability (Livingston et al. 2017; Stith et al. 2018), analyzed a smaller study sample (Stith et al. 2018) or had a shorter study period (Bradford and Bradford 2016).

### Summary of findings

#### Opioid overdose mortality

**Fig. 2:**
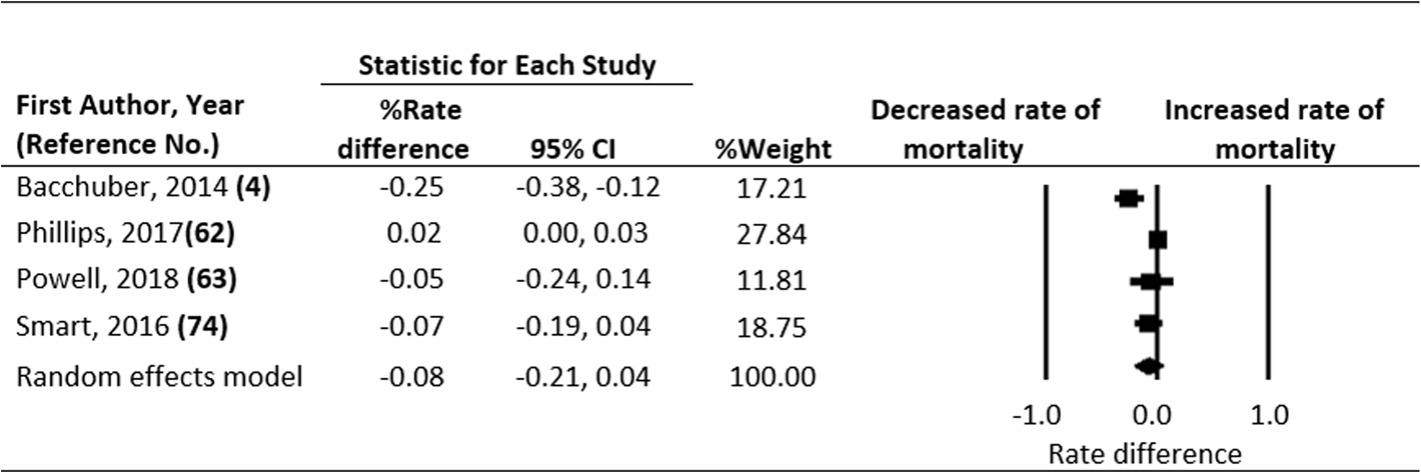
Forest Plot, Summary Percent Rate Differences (RD) and 95% Confidence Intervals (CI) of Opioid-related Mortality Associated with Medical Marijuana Laws in the U.S. The Diamond Indicates the Summary Percent RD. Horizontal Bars Indicate the 95% CI. Heterogeneity: Q statistic: 24.080, df = 4, P = 0.000, I^2^ = 83.389

Of the 4 studies that examined the association between MMLs and opioid overdose mortality, 1 reported a statistically significant reduction in mortality (Bachhuber et al. 2014), 1 reported a statistically significant increase in mortality (Phillips and Gazmararian 2017), and 2 found reductions that were not statistically significant (Powell et al. 2018; Smart 2016). Although the latter two studies found no overall significant impact, Smart (2016) reported significantly lower opioid overdose mortality among adults aged 45–64 years in MML states compared to non-MML states. Similarly, Powell et al. (2018) found a statistically significant 27% reduction in opioid overdose mortality in states with active and legal marijuana dispensaries compared to those without. Effect estimates showed a presence of significant heterogeneity (Q statistic = 24.080, df = 4, *P* < 0.001; I^2^ = 83.389). Random effects modeling based on pooled data from the 4 studies indicates that implementation of MMLs was associated with a statistically non-significant 8% reduction in opioid overdose mortality [95% confidence interval (CI) = − 0.21 to 0.04; Fig. 2]. Rosenthal’s fail-safe N did not indicate any major publication bias. Livingston et al. (2017) assessed the impact of legalizing marijuana for recreational use in Colorado and found that the policy change contributed to a 7% reduction in opioid overdose mortality (95% CI = − 0.128 to − 0.002).

#### Opioid prescriptions

**Fig. 3:**
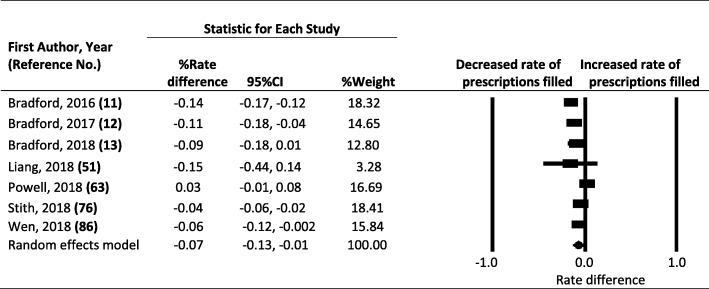
Forest Plot, Summary Percent Rate Differences (RD) and 95% Confidence Intervals (CI) of Opioid Prescriptions Filled Associated with Medical Marijuana Laws in the U.S. The Diamond Indicates the Summary Percent RD. Horizontal Bars Indicate the 95% CI. Heterogeneity: Q statistic: 70.276, df = 6, P = 0.000, I^2^ = 91.462

Of the 7 studies assessing the association between MMLs and opioid prescriptions, 4 reported that implementation of MMLs was attributed to a significant decline in prescription opioids dispensed (Bradford and Bradford 2017; Bradford and Bradford 2016; Stith et al. 2018; Wen and Hockenberry 2018), 2 reported declines that were not statistically significant (Bradford et al. 2018; Liang et al. 2018), and 1 found a statistically non-significant increase (Powell et al. 2018). Effect estimates showed a presence of heterogeneity (Q statistic = 70.276, df = 6, P < 0.001; I^2^ = 91.462). Rate differences ranged from − 15 to + 3% (Fig. 3). Pooled data indicate that implementation of MMLs was associated with a 7% reduction in prescription opioids dispensed (95% CI = − 0.13 to − 0.01; Fig. 3). Rosenthal’s fail-safe N did not indicate any major publication bias. Wen and Hockenberry (2018) also assessed the effect of state recreational marijuana laws on opioid prescribing in Medicaid and managed care enrollees and found that legalizing marijuana for recreational use was associated with a 6% reduction in the opioid prescription rate (95% CI = -0.122 to − 0.006).

#### Opioid-related hospitalizations

Two studies assessed the impact of state MMLs on opioid-related hospitalizations (Shi 2017; Powell et al. 2018). Shi (2017) found that implementation of MMLs was associated with a 23% reduction in hospitalizations related to opioid abuse or dependence (95% CI = -0.41 to − 0.07) and a 13% reduction in prescription opioid overdoses (95% CI = -0.25 to − 0.02). Powell et al. (2018) analyzed 2 sets of data: 1999–2010 and 1999 to 2013. In the first dataset, Powell et al. (2018) found no significant reductions in opioid-related hospitalizations associated with implementation of state MMLs but reported a significant reduction in opioid-related hospitalizations associated with MMLs allowing active and legal marijuana dispensaries. In the second dataset, Powell et al. (2018) found that MMLs, regardless of the availability of active and legal marijuana dispensaries, were not associated with opioid-related hospitalizations.

#### Nonmedical use of prescription opioids

Three studies assessed the association of MMLs with nonmedical use of prescription opioids (Cerda et al. 2018; Powell et al. 2018; Wen et al. 2015). Wen et al. (2015) reported that MMLs had no discernible impact on the prescription painkiller (including opioids) misuse among adolescents and adults. Cerda et al. (2018) studied a nationally representative sample of adolescents and concluded that MML enactment was associated with increases in nonmedical use of prescription opioids among 12th graders. Powell et al. (2018) found no association between MMLs and nonmedical use of prescription opioids.

#### Other outcomes

Kim et al. (2016) assessed the association of MMLs with opioid positivity among drivers involved in fatal motor vehicle crashes in 18 states with high drug testing rates. Overall, they found no association but reported a significant decrease in opioid positivity among drivers aged 21–40 years (Kim et al. 2016). Bradford and Bradford (2016, 2017) estimated national overall savings of $165.2 million per year in the Medicare program when states implemented MMLs and savings of over $1 billion in fee-for-service Medicaid programs had all states implemented MMLs.

## Discussion

In this study, we found no conclusive evidence that MMLs are associated with reductions in prescription opioid overdose mortality. Although one widely cited study found a 25% reduction in overdose mortality (Bachhuber et al. 2014), only one subsequent study reported a significant, albeit much smaller, reduction associated with recreational marijuana legalization in Colorado (Livingston et al. 2017). Similarly, Powell et al. (2018) found no significant overall effect of MMLs on opioid overdose mortality but reported a 27.2% reduction in opioid overdose mortality in states with active and legal marijuana dispensaries. These findings highlight the potentially important role of the presence of active and legal dispensaries beyond MML enactment and implementation. More research is needed to assess the specific features of state marijuana laws on opioid overdose mortality and other opioid-related health outcomes. In particular, evidence from longitudinal studies would be valuable for better understanding the impact of marijuana laws on the opioid epidemic as more states legalize marijuana for medical and recreational use (National Conference of State Legislation 2019).

Findings from this systematic review show that MMLs are associated with a modest reduction in opioid prescriptions. Specifically, implementation of MMLs is associated with a 7% reduction in opioid prescriptions. The magnitude of the effect of state MMLs on opioid prescriptions is rather modest, suggesting that marijuana is unlikely a major substitute for prescription opioids. Previous surveys conducted in the United States (Boehnke et al. 2016; Corroon et al. 2017; Reiman et al. 2017; Sexton et al. 2016), Canada (Lucas and Walsh 2017; Lucas et al. 2012) and Israel (Haroutounian et al. 2016) have reported rates of up to 64% reduction in opioid prescriptions. The discrepancy is due in part to study design differences and measurement ascertainment. Our review included only studies that analyzed objectively measured opioid-related outcomes such as overdose mortality and prescriptions dispensed.

There are at least two plausible explanations for the modest reduction in opioid prescriptions associated with state MMLs. First, marijuana may be perceived as a safer substitute associated with a lower risk of overdose and less side effects (Zaller et al. 2015), greater pain reduction (Andreae et al. 2015; National Academies of Sciences Engineering and Medicine 2017; Whiting et al. 2015), and potential to alleviate opioid-related addiction (Lucas et al. 2012). Second, the cannabinoid receptor system and the opioid receptor system appear to have anatomical and biochemical similarities (Bushlin et al. 2010). Activation of the cannabinoid receptors increases the analgesic effect of marijuana through direct inhibition of acetylcholine, dopamine and serotonin (Sohler et al. 2018) as well as indirect stimulation of opioid receptors thereby modulating spasticity, motor function, and pain (Borgelt et al. 2013). Further, preclinical trials have shown marijuana to have independent analgesic capability (Hayes and Brown 2014) that is augmented in the presence of an opioid (Abrams et al. 2011). The extent to which marijuana can provide enough pain control as an adjuvant therapeutic with reduced prescription opioid dosages merits further investigation.

The presumed benefit of legalizing marijuana in reducing opioid-related harms should be weighed against potential unfavorable externalities. For example, results from controlled trials have found that patients with chronic pain who use marijuana have more severe pain, tend to use more prescription opioids and higher doses (Degenhart et al. 2015; Hefner et al. 2015). In addition, another study reported that marijuana users were much more likely to develop opioid use disorder (Olfson et al. 2018). It is evident that the prevalence of marijuana use among adolescents is higher in states with MMLs compared to those without (Hasin et al. 2015; Stolzenberg et al. 2016) and illicit marijuana use is associated with greater risk of opioid misuse among adolescents (Cerda et al. 2018; Fiellin et al. 2014). Further, marijuana arrests and treatment admissions to rehabilitation facilities among young adult males are higher in MML states compared to non-MML states (Chu 2014). Although medical marijuana is authorized for specific medical conditions, the increased availability of marijuana (Freisthler and Gruenewald 2014) combined with lower perception of marijuana risk (Schuermeyer et al. 2014) may lead to other public health problems such as drugged driving (Brady and Li 2013; Guenzburger and Masten 2013), cognitive impairment (Volkow et al. 2014), acute intoxication (Davis et al. 2016), dependence, psychosis (Patel et al. 2016), and pulmonary disorders (Wilkinson et al. 2016).

This systematic review has several notable limitations. First, most of the studies included in this review were based on state-level data, making their findings susceptible to the ecological fallacy (i.e., not directly translatable to opioid-related outcomes on individual level). Second, studies included in this review varied in designs and analytical approaches and adjusted for different covariates, which may contribute to the inconsistent findings. Although most studies included in this review controlled for time-varying and fixed state effects such as population, education, racial composition, and prescription drug monitoring program, confounding from unmeasured variables, such as naloxone distribution and access to medication assisted treatment program, remains a concern (Hall et al. 2018). Third, studies included in the meta-analyses are relatively few and showed significant heterogeneity. Therefore, evidence from this review should be viewed as preliminary and interpreted with caution. Finally, the surge of illicit fentanyl after 2014 is a major driver of the opioid epidemic in recent years. Therefore, studies assessing the impact of MMLs on opioid overdose mortality using data for 2014 and after, such as the report by Shover et al. (2019), can be seriously confounded by the overriding role of fentanyl and analogs.

## Conclusions

Legalizing marijuana might contribute to a modest reduction in opioid prescriptions. Evidence about the effect of marijuana legalization on opioid overdose mortality is inconsistent and inconclusive. If any, the effect of state marijuana laws in reducing opioid overdose mortality appears to be rather small and limited to states with operational marijuana dispensaries. Evidence on other opioid related-outcomes, such as hospitalizations and nonmedical use, is sparse. It remains unclear whether the presumed benefit of legalizing marijuana in reducing opioid-related harms outweighs the policy’s externalities, such as its impact on mental health and traffic safety.

## Data Availability

Data analyzed in the current study were abstracted from publicly available studies and are available from the corresponding author upon request.

## Abbreviations

CI: Confidence interval
MM: Medical marijuana
MML: Medical marijuana law
PDMP: Prescription drug monitoring program
RD: Risk difference
